# The effectiveness of psychosocial interventions in war-traumatized refugee and internally displaced minors: systematic review and meta-analysis

**DOI:** 10.1080/20008198.2017.1388709

**Published:** 2017-11-07

**Authors:** Agnes Nocon, Rima Eberle-Sejari, Johanna Unterhitzenberger, Rita Rosner

**Affiliations:** ^a^ Department of Psychology, Catholic University Eichstätt-Ingolstadt, Eichstätt, Germany

**Keywords:** Refugees, displaced, war, children, adolescents, psychotherapy, psychosocial interventions, refugiados, desplazado, guerra, niños, adolescentes, psicoterapia, intervenciones psicosociales, 难民, 失所, 战争, 儿童, 青年, 心理治疗, 心理社会干预, • We performed a review and meta-analysis on intervention effects in forcibly displaced war-traumatized minors.• The field is marked by the use of weak research designs and non-evidence-based interventions.• The majority of studied interventions show null effects or are non-significant.• Evidence-based interventions like cognitive behavioural treatment or interpersonal therapy showed promising but inconsistent results. Replication studies are missing.• The effect sizes stay behind the effects reported in children and adolescents from the general population.

## Abstract

**Background**: The United Nations reported that in 2016 over 65 million people worldwide have forcibly left home. Over 50% are children and adolescents; a substantial number has been traumatized and displaced by war.

**Objective**: To provide an overview of the effectiveness of psychosocial interventions in this group we conducted a narrative review and a meta-analysis of intervention studies providing data on posttraumatic stress symptoms (PTSS), depression, anxiety, grief, and general distress.

**Method**: We searched PILOTS, MEDLINE, WoS, Embase, CENTRAL, LILACS, PsycINFO, ASSIA, CSA, and SA for studies on treatment outcomes for war-traumatized displaced children and adolescents. Between-group effect sizes (ES) and pre-post ES were reconstructed for each trial. Overall pre-post ES were calculated using a random effects model.

**Results**: The narrative review covers 23 studies with a variety of treatments. Out of the 35 calculated between-group ES, only six were significant, all compared to untreated controls. Two of them indicated significant adverse effects on symptoms of general distress or depression. When calculating pre-post effect sizes, the positive between-group results of cognitive behavioural therapy (CBT) and interpersonal therapy (IPT) were reproduced and singular other treatments showed significant positive effects. However, the mean pre-post effects for PTSS and depression could not be interpreted due to the high heterogeneity of the included studies (PTSS: ES = 0.78; I^2^ = 88.6%; depression: ES = 0.35; I^2^ = 93.1%). Only the mean pre-post effect for seven active CBT treatment groups for depression (ES = 0.30, 95% CI [0.18, 0.43]) was interpretable (Q = 3.3, *df* = 6, *p* = .77).

**Conclusion**: Given the large number of children and adolescents displaced by war there were regrettably few treatment studies available, and many of them were of low methodological quality. The effect sizes lagged behind the effects observed in traumatized minors in general, and often were small or non-significant. However, CBT and IPT showed promising results that need further replication.

## Abstract (es)

**Planteamiento:** Las Naciones Unidas informaron de que en 2016 más de 65 millones de personas en todo el mundo han tenido que abandonar a la fuerza sus hogares. Más del 50% son niños y adolescentes. Muchos de ellos han sido traumatizados y desplazados por la guerra.

**Objetivo:** Con el fin de proporcionar una visión general de la eficacia de las intervenciones psicosociales en este grupo, se realizó una revisión narrativa y un metanálisis de los estudios de intervención que proporcionaban datos sobre síntomas de estrés postraumático (SEPT), depresión, ansiedad, duelo y malestar general.

**Método:** Buscamos en PILOTS, MEDLINE, WoS, Embase, CENTRAL, LILACS, PsycINFO, ASSIA, CSA y SA estudios sobre resultados de tratamiento para niños y adolescentes traumatizados y desplazados por la guerra. Se reconstruyeron los tamaños del efecto (TE) entre los grupos y los TE pre-post para cada ensayo. Los TE generales pre-post fueron calculados usando un modelo de efectos aleatorios.

**Resultados:** La revisión narrativa consta de 23 estudios con una amplia variedad de tratamientos. De los 35 TE entre grupos calculados, sólo 6 fueron significativos, todos en comparación con controles no tratados. Dos de ellos indicaban efectos adversos significativos en los síntomas de malestar general o en los síntomas de depresión. Al calcular los TE pre-post, se reprodujeron los TE entre grupos positivos de la terapia cognitivo-conductual (TCC) y la terapia interpersonal (TIP) y otros tratamientos singulares mostraron efectos positivos significativos. Sin embargo, los efectos promedio pre-post para SEPT y depresión no pudieron interpretarse debido a la alta heterogeneidad de los estudios incluidos (SEPT: TE = 0.78, I^2^ = 88.6%, depresión: TE = 0.35; I^2^ = 93.1%). Sólo se pudo interpretar el efecto promedio pre-post para 7 grupos activos de tratamiento con TCC para la depresión, TE = 0.30, IC del 95% [0.18, 0.43] (Q = 3.3, *df* = 6, p = 0.77).

**Conclusión:** Dado el gran número de niños y adolescentes desplazados por la guerra, lamentablemente existen pocos estudios de tratamiento disponibles y muchos de ellos de baja calidad metodológica. Los tamaños del efecto se quedaron a la zaga de los efectos observados en los menores traumatizados en general, y a menudo eran pequeños o no significativos. Sin embargo, la TCC y la TIP mostraron resultados prometedores que necesitan replicarse más.

## Abstract (zh)

**背景：**联合国报道在2016年全世界超过650万人口被迫离开家园，其中超过50%是儿童和青少年，大多数都经历了创伤，并因为战争流离失所。

**目标：**为了提供一个在这个群体里心理社会干预的总结，我们进行了一个叙述性综述和干预研究的元分析，提供了关于创伤后应激症状、抑郁、焦虑、哀痛和一般痛苦的数据。

**方法：**我们在PILOTS, MEDLINE, WoS, Embase, CENTRAL, LILACS, PsycINFO, ASSIA, CSA 和 SA数据库检索了战争创伤流离失所的儿童和青少年的治疗结果研究。组间效应量（ES）和前-后ES针对每一个试验进行了重组。总体的前-后ES使用随机效应模型进行计算。

**结果：**这个叙述性综述涵盖了23个关于多种治疗方法的研究。在35个计算出来的并和未治疗控制组进行对比的组间ES中，只有6个是显著的。2个研究报告了表现对一般痛苦或者抑郁症状的显著负面影响。计算前-后效应量时，CBT和人际关系治疗（IPT）的组间阳性结果得到了重复，其他治疗方法单一地显示了显著的阳性结果。但是PTSD和抑郁的平均前-后效应量不能被解释，因为所包括的研究的高异质性(PTSD: ES = 0.78; I2 = 88.6%; 抑郁: ES = 0.35; I2 = 93.1%)。只有7个抑郁CBT治疗组的平均前-后效应量（ES = 0.30, 95% CI [0.18, 0.43]）是可以解释的（Q = 3.3, df = 6, p = 0.77）。

**结论：**大量的儿童青少年在战争中流离失所，遗憾的是相关治疗研究太少了，其中许多研究的研究质量较低。效应量比在创伤青少年中观察总体效应低，并且经常较小或者不显著。但是，CBT和IPT显示了有希望的结果，需要更多的重复研究。

## 1. Introduction

The last few years have seen a fourfold increase in mass displacement resulting from wars and conflicts worldwide. In 2016, over 65 million people worldwide were forced to leave their homes (UNHCR, 2017). Children below 18 years make up 51% of this population, a rate that has been rising constantly. Although forced displacement and its causes constitute sufficiently severe stressors to cause suffering in anyone, children and adolescents are particularly vulnerable. A substantial body of literature documents the accumulation of diverse traumata and psychosocial risk factors in displaced war-affected minors (Jensen & Shaw, 1993; Kletter et al., 2013), leading to large rates of psychological problems (Lustig et al., 2004) and subsequent complications (e.g. Vervliet, Lammertyn, Broekaert, & Derluyn, 2014). For example, several research groups report prevalence rates for mental health problems of up to 80% in unaccompanied asylum-seeking children, with posttraumatic stress disorder (PTSD), depression, and anxiety disorders (Bronstein, Montgomery, & Dobrowolski, 2012; Bronstein, Montgomery, & Ott, 2013; Huemer et al., 2009; Jakobsen, Demott, & Heir, 2014), as well as traumatic grief and conduct problems (Betancourt, Newnham, Layne, et al., 2012b), the latter being the most frequent diagnoses.

Overall, there is a consensus among health care professionals that these young people require particular assistance, but a number of obstacles hamper the delivery of psycho-social support: there are not enough trained psychotherapists available; access to effective treatment for refugees is limited for a number of reasons like geographical location or finances; the concept of psychotherapy and motivation for it differs across cultures; and there is no clear-cut recommendation on whether western evidence-based treatments are effective and applicable in this group.

Recent meta-analyses on PTSD treatment in children and adolescents after various kinds of trauma show converging evidence with overall effect sizes of g = 0.83 and 0.89 when compared to waitlist and of g = 0.41 and 0.45 when compared to active controls (Gutermann et al., 2016; Morina, Koerssen, & Pollet, 2016; respectively). Effect sizes for depression were 0.60 and for anxiety 0.67 when compared to waitlist, and 0.37 and 0.42 in comparison to active controls (Gutermann et al., 2016). Both meta-analyses, however, report considerable heterogeneity in their study, rendering the interpretation of the results difficult.

Contrary to the emerging evidence for children and adolescents with PTSD after various traumatic events, the evidence-based guidance for the treatment of war-affected displaced minors, though increasingly necessary, remains scarce.

Some authors provide a first overview of the field. Tyrer & Fazel (2014) reviewed health interventions for displaced children in school and community settings, identifying 21 studies. Due to the considerable variation in the delivered treatments, the authors refrained from calculating an overall effect size for controlled comparisons. Cohen’s *d* were reported for two studies on depression (*d* = 0.57 and 0.93), two on anxiety (*d* = 0.64 and 0.93), three on PTSD-symptoms (*d* = 0.31–0.92), and six studies on other conditions like functional impairment and behavioural problems (*d* = 0.32–0.79), without specifying the respective control groups.

A recent meta-analysis on war-affected children and adolescents in low- and middle-income countries (Morina, Malek, Nickerson, & Bryant, 2017) identified 21 randomized controlled trials and reported pre-post effect sizes of g = 1.15 and a medium effect size of g = 0.53 compared to waitlist for posttraumatic stress symptoms. Effects on depressive symptoms were considerably lower, with g = 0.30 and g = 0.25, respectively. However, substantial heterogeneity impairs the interpretation of these overall effect sizes. The study sample in this publication consisted of children with mixed traumata, including a number of original studies on child soldiers who were perpetrators and victims at the same time. Their treatment might need to be specifically adapted (Betancourt, Newnham, Brennan, et al., 2012a, showed that treatment effects in abducted and non-abducted children were different) and should possibly include reconciliation.

Summarizing the above, previous reviews were based on samples of young people who were either affected by war or displaced, but not necessarily both. We aim to detect the efficacy of any putatively useful psychosocial interventions for forcedly displaced minors, as these children constitute a group with particular needs. To this end, we build on our previous work on displaced minors (Eberle-Sejari, Nocon, & Rosner, 2015), focus specifically on those who were traumatized by war, and expand on it by including a meta-analytic approach. To include all identified publications irrespective of their study design, we will also consider pre-post effect sizes.

## 2. Methods

### 2.1. Eligibility criteria

To detect any psychosocial intervention for the affected population, including primary prevention programmes, we chose broad search criteria:

- Participants: refugees and internally displaced persons with direct war-related trauma exposure at the age of 18 years or under. Child soldiers were excluded.
- Interventions: any intervention within the health care system intended to alleviate symptoms of trauma-related disorders.
- Comparators: all comparators were eligible.
- Outcomes: diagnosis and symptoms of PTSD, depression, anxiety, general distress, and complicated grief, as measured by some form of structured interview or questionnaire.
- Study design: randomized controlled trials (RCT), controlled trials (CT), and uncontrolled pre-post studies. If they provided any information about negative effects, we aimed to include case studies and case reports in the narrative review.

There were no language restrictions. We planned to identify all possible studies and search for translators in cases where no member of the research team could read the paper. We aimed to include all relevant studies, regardless of their publication status, to avoid publication bias. Unpublished and ongoing studies were sourced and included; to this end, potential authors were contacted. Conference abstracts were not included.

### 2.2. Literature search

We searched for published and unpublished studies in the following databases: Pilots, Medline (through the PubMed interface), Web of Science Core Collection, Embase, Central, Lilacs, PsycInfo, ASSIA, CSA, and SA (date of last search: 28 June 2017). Snowballing techniques included searching websites, journal hand searches, contacting authors and colleagues, and reference list checking. Searches were not restricted by language or publication date.

Keywords were *child, adolescent, war, refugee, therapy, treatment, psychotherapy, treatment outcomes*, and *emotional trauma*, according to the thesaurus and truncation options of the respective database.

### 2.3. Data collection

One author screened titles and abstracts (AN). Two other authors independently rated study characteristics (RES, JU). Each of them checked the other’s work to ensure accuracy. Coded study characteristics were: (1) residence status (refugee, internally displaced); (2) group assignment (randomized, matched, convenience, no control group); (3) age range; (4) percentage of female participants; (5) exclusion of severe cases (suicidal, severe symptomatology, psychosis, language, other); (6) status of providers with regard to training; (7) type of intervention; (8) dose of intervention (in 50 minute sessions); (9) handling of drop-outs (intent to treat analysis, last observation carried forward, completer analysis); (10) number and duration of follow-up assessments; (11) control condition (waitlist/no treatment, unspecific treatment like support or counselling, psychotherapy). In cases where the raters were discordant on any characteristic, two other authors recoded the information (AN, RR). One author extracted data on statistical measures (AN). Primary outcomes were changes in symptom measures such as PTSD, depression, anxiety, general distress, or complicated grief.

As a major part of identified studies was of low study quality (no control group, no quantitative data), we indicated risk of bias only via the broad category of study design (RCT, CT, uncontrolled pre-post study). We aimed to use all available studies at this very early stage of research in the field, and focused the meta-analysis on within-group treatment effects. To appraise the validity of the pre-post effects, we additionally considered unexplained drop-out rates and whether intent-to-treat analyses were used.

### 2.4. Statistics

For controlled trials, between-group effect sizes (standardized mean differences; SMD) were reconstructed as Cohen’s *d* with pooled standard deviation. To detect all putatively positive effects regardless of study design, pre-post effect sizes were reconstructed for all eligible treatment conditions (i.e. from control groups and uncontrolled studies also). In these cases, standardized mean changes (SMCs) were computed as raw score standardized mean changes (Morris & DeShon, 2002). In the paper by Onyut et al. (2005) the depression effect size was calculated using the marginal odds ratio (Zou, 2007), missing values were imputed using follow-up data on *n* = 6, and 0.5 was added to every cell of the contingency table to address the problem of empty cells (Cox, 1970). All effect sizes were corrected for sample size.

We used the random effects model for data synthesis as we did not expect all included studies to share one true effect size (samples may, for example, differ with regard to types of traumatization, age ranges, and treatments). I^2^ was used as the measure for consistency and the Q-test for heterogeneity. To test for small study effects that can indicate publication bias we used regression tests (Egger, Smith, Schneider, & Minder, 1997). The effect of residence status as moderator was assessed using a mixed-effects model for subgroup comparisons (Borenstein, Hedges, Higgins, & Rothstein, 2009) and the Q-test for moderator variables was reported (QM). We refrained from further moderator analyses (e.g. regarding control conditions) as the small number of studies only allowed for moderator analysis with dichotomized moderators.

All statistics were computed using the statistics software R 3.2.2 (R Core Team, 2015) including the metafor package (Viechtbauer, 2010).

## 3. Results

### 3.1. Study selection

**Figure 1.**
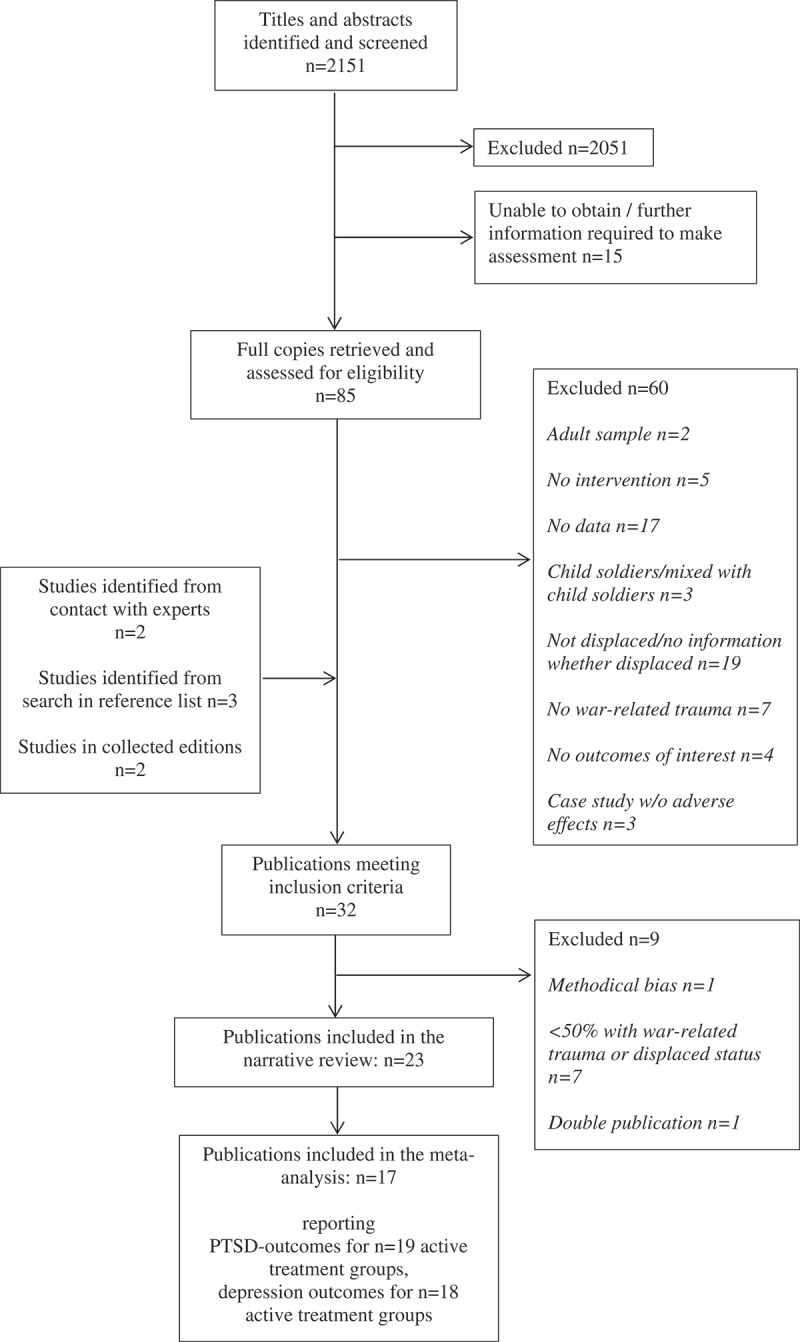
PRISMA flow diagram of the literature search.

Twenty-three publications met the inclusion criteria (Figure 1) and were included in the narrative review. Two studies were part of doctoral theses (Ooi, 2012, pilot study; Schauer, 2008), three were short reports in an omnibus volume (Išpanović-Radojković, Petrović, Davis, Tenjović, & Minčić, 2002; Šehović, 2002; Šestan, 2002), and 17 were original articles. All identified publications were in English.

Nine studies were excluded although they initially fulfilled eligibility criteria. The pilot study of Sadeh and colleagues (2008), reported baseline data in Israeli children who were internally displaced during the second Lebanon–Israel war, with post-assessment after the end of war and the return home. It was excluded for methodological bias, as the end of the war may account for any effects detected in this study. Five publications were excluded because displaced persons represented a minority (up to 31%) in the study sample (Diab, Peltonen, Qouta, Palosaari, & Punamäki, 2015; Kangaslampi, Punamäki, Qouta, Diab, & Peltonen, 2016; Newnham et al., 2015; Punamäki, Peltonen, Diab, & Qouta, 2014; Tol et al., 2008). Two studies were excluded because only a minority (up to 35%) reported war-related traumata (Unterhitzenberger et al., 2015; Ruf et al., 2010). One study (Ispanovic-Radojkovic, 2003) was a double publication with Ispanovic-Radojkovic et al. (2002).

### 3.2. Part 1: narrative review

#### 3.2.1. Study characteristics

All 23 identified publications were published in the last 15 years. The studies included children of diverse psychopathological status: children stemming from a war-affected area irrespective of their individual distress to children with full-blown PTSD diagnosis. Sample sizes ranged from *N* = 4 (Ooi, 2012) to *N* = 399 (Tol et al., 2012).

Eight studies used a randomized controlled design, six used non-randomized control groups, and eight were uncontrolled pre-post studies (see Table 1, where the studies are grouped accordingly). Among the eight RCTs, three studies did not provide full details of the randomization method (Kalantari, Yule, Dyregrov, Neshatdoost, & Ahmadi, 2012; Lange-Nielsen et al., 2012; Tol et al., 2012) and three used small experimental and control groups (*n* < 30) (Catani et al., 2009; Schauer, 2008; Schottelkorb, Doumas, & Garcia, 2012), which means that the randomization may not have been successful. However, no significant group differences on putatively relevant characteristics were detected in either of these studies. Among all studies with control groups, only five studies reported blinding of raters to participant group allocation (Betancourt et al., 2012a; Catani et al., 2009; Dybdahl, 2001; Ellis et al., 2013; Schauer, 2008). The success of the blinding procedures was not tested in any of these studies.

Attrition was fully reported or did not occur in 15 out of 23 studies. In three studies, drop-outs were not recorded (Išpanović-Radojković et al., 2002; Šehović, 2002; Šestan, 2002). Five studies reported drop-outs but gave no reasons for them (Dybdahl, 2001; Kalantari et al., 2012; Lange-Nielsen et al., 2012; O’Shea, Hodes, Down, & Bramley, 2000; Schottelkorb et al., 2012). Attrition rates ranged from 2.9% (Gupta & Zimmer, 2008) to 50% (O’Shea et al., 2000). O’Shea and colleagues did not report the reasons for the high attrition. Two studies reported intent-to-treat analyses (Betancourt et al., 2012a; Onyut et al., 2005).

Two studies reported treatment fidelity problems. In the study of Schottelkorb and colleagues (2012), parents did not participate in the therapies as expected. In the study of Schauer (2008), trained teachers supported families beyond the scope of the manual.

#### 3.2.2. Treatment characteristics

Thirteen out of 23 studies examined the effect of evidence-based treatments, such as interpersonal therapy (IPT) (Betancourt et al., 2012a), strict cognitive behavioural therapy (CBT) (Catani et al., 2009; Onyut et al., 2005; Pfeiffer & Goldbeck, 2017; Schauer, 2008; Schottelkorb et al., 2012), eclectic CBT with other elements (Ehntholt, Smith, & Yule, 2005; Möhlen, Parzer, Resch, & Brunner, 2005; Ooi, 2012; Ooi et al., 2016; Šehović, 2002; Tol et al., 2012), or eye movement desensitization and reprocessing (EMDR; within a psychodynamic therapy) (Oras, De Ezpeleta, & Ahmad, 2004).

Additionally, the following non-evidence based treatments were examined either in the experimental or the control condition: creative play (Betancourt et al., 2012a), child-centred play therapy (Schottelkorb et al., 2012), writing intervention (writing for recovery; Kalantari et al., 2012; Lange-Nielsen et al., 2012), meditation and relaxation techniques (Schauer, 2008), crisis intervention (Thabet, Vostanis, & Karim, 2005), psychosocial support (Šestan, 2002), psychoeducation alone (Thabet et al., 2005), a systemic approach with preventive skill building (Ellis et al., 2013), psychosocial support combined with medical care (Dybdahl, 2001), or mixed interventions/eclectic (Fazel, Doll, & Stein, 2009; O’Shea et al., 2000). One study examined the effects of a school educational programme (Gupta & Zimmer, 2008). The four-week programme was provided by trained teachers, and its additive psychological parts consisted of structured trauma-focused activities twice a week (mainly psychoeducation), play, and expression techniques (UNESCO, 2000; UNESCO/FAWE, 1999). One study presented a preventive school programme including social games and sociotherapy groups on a voluntary basis (Išpanović-Radojković et al., 2002).

The treatment dose varied from 1.5 hours to weekly sessions throughout an entire school year. In 13 studies, the interventions were provided in a school setting (Ehntholt et al., 2005; Ellis et al., 2013; Fazel et al., 2009; Išpanović-Radojković et al., 2002; Kalantari et al., 2012; Lange-Nielsen et al., 2012; O’Shea et al., 2000; Ooi, 2012; pilot; Ooi et al., 2016; Schauer, 2008; Schottelkorb et al., 2012; Thabet et al., 2005; Tol et al., 2012). In 12 studies, trained lay persons (e.g. teachers, social workers) provided the main intervention (Betancourt et al., 2012a; Catani et al., 2009; Dybdahl, 2001; Ellis et al., 2013; Gupta & Zimmer, 2008; Išpanović-Radojković et al., 2002; Möhlen et al., 2005; Pfeiffer & Goldbeck, 2017; Schauer, 2008; Schottelkorb et al., 2012; Thabet et al., 2005; in the psychoeducation group; Tol et al., 2012).

#### 3.2.3. Between-group effect sizes in studies using control groups

To enhance the comparability of the achieved treatment effects, we report between-group effect sizes according to broad categories of the used control conditions: no treatment/waitlist, unspecific treatment, psychotherapy.

Table 1 summarizes the between-group effect sizes for PTSD and depression, together with their respective 95% confidence intervals. Two studies using CBT reported medium and large effects on PTSD compared to untreated controls (SMD = 0.88, Ehntholt et al., 2005; SMD = 0.37 in the group of girls, Tol et al., 2012; see Table 1). Any other effects on PTSD were null or not significant, that is, the confidence intervals included the zero. Regarding treatment effects on depression, IPT had large positive effects compared to waitlist controls, but only in girls (SMD = 1.06, Betancourt et al., 2012a), while writing for recovery had large adverse effects (SMD = −1.25, Lange-Nielsen et al., 2012) which had disappeared by follow-up. Any other reported effects were null or small, and not significant.

**Table 1.** Study characteristics and between-group effect sizes (standardized mean differences) by study design.

									SMD experimental vs control condition for PTSD (95% CI)				SMD experimental vs control condition for depression (95% CI)			
Publication	Age	*N*	Country of origin	Pathology at inclusion	Experimental condition	Intervention dose^#^	Control condition	Instrument PTSD	vs no treatment	vs unspecific treatment	vs psychotherapy	Instrument Depression	vs no treatment	vs unspecific treatment	vs psychotherapy	Instruments other outcomes
**Randomized controlled trials**																
Betancourt et al. (2012a)	14–17	314	Uganda	Significant depression symptoms	IPT	21–28	1) CP; 2) WL	–	–	–	–	APAI	−0.02 [−0.64, 0.60] (boys)$; 1.06 [0.61, 1.52] (girls)$	NA	–	–
Catani et al. (2009)	8–14	31	Sri Lanka	Preliminary PTSD diagnosis (3 weeks after tsunami)	KIDNET	7.2–10.8	Med-Relax	UCLA	–	0.01 [−0.68, 0.71]	–	–	–	–	–	distress: 5 self-designed questions related to problems in functioning
Dybdahl (2001)	5–6	87	Bosnia and Herzegovina	Any children from displaced mothers	Parent psychosocial support + medical care	20	Med	–	–	–	–	BDI interview	–	−0.07 [−0.53, 0.38]	–	distress: total score of mothers’ rating of children’s problems
Kalantari et al. (2012)	12–18	61	Afghanistan	Highest traumatic grief score	Writing for Recovery	1.8	NA	–	–	–	–	–	–	–	–	grief: TGI
Lange-Nielsen et al. (2012)	12–17	169	Gaza	Any children from refugee camps	Writing for Recovery	1.8	WL	CRIES-13	0.11 [−0.25, 0.47]	–	–	DSRS	−1.25 [−1.64, −0.86]	–	–	Anx: RCMAS
Schauer (2008)	11–15	47	Sri Lanka	Full PTSD diagnosis	KIDNET	10.8	Med-Relax	CAPS-CA	–	0.06 [−0.44, 0.56]	–	MINI	–	0.22 [−0.28, 0.72]	–	–
Schottelkorb et al. (2012)	6–13	31	Predominantly crisis regions	PTSD symptoms	CCPT	11.1	modified TF-CBT	UCLA	–	–	0.26 [−0.49, 1.01]	–	–	–	–	–
Tol et al. (2012)	9–12	399	Sri Lanka	Screened as being traumatized	CBT	15	WL	CPSS	0.00 [−0.26, 0.26] (boys); 0.37 [0.05, 0.69] (girls)	–	–	DSRS	−0.04 [−0.30, 0.21] (boys); 0.15 [−0.17, 0.48] (girls)	–	–	Anx: SCARED-5
**Controlled trials**																
Ehntholt et al. (2005)	11–15	26	Mixed	Psychological or behavioural difficulties	TRT	7.2	WL	CRIES-13	0.88 [0.07, 1.69]	–	–	DSRS	0.26 [−0.52, 1.05]	–	–	anxiety: RCMAS, distress: SDQ (teacher)
Ellis et al., 2013	11–15	30	Somalia	TG: signs of emotion dysregulation; CG: no signs of emotion dysregulation	TST	tier 3: 23; tier 4: n.a.	preventive skill building	UCLA	–	−0.01 [−0.60, 0.57]	–	DSRS	–	−0.26 [−0.85, 0.32]	–	–
Fazel et al. (2009)	5–17	141	Mixed	Emotional and behavioural problems	Multilevel: counselling of teachers, direct diverse techniques	2–36	Untreated ethnic minority and indigenous children	–	–	–	–	–	–	–	–	distress: SDQ (teacher)
Ispanovic et al. (2002)	15–18	158	Serbia	Any adolescents	Youth Club	24	Untreated	–	–	–	–	YSF	favourable for experimental condition	–	–	anxiety: YSF
Ooi et al. (2016)	11–17 and outside	82	Africa and other	Mild to moderate PTSD symptoms	TRT	9.6	WL (was treated postintervention)	CRIES-13	−0.02 [−0.44, 0.40]	–	–	DSRS	0.03 [−0.39, 0.44]	–	–	distress: SDQ (parent and teacher)
Šehović (2002)	6.5–16	122	Bosnia and Herzegovina	Traumatized	CBT and other	36	eclectic	PTSD-RI, IES-R, PTSD-questionnaire for children	–	–	favourable for experimental condition	–	–	–	–	–
Šestan (2002)	6–7	96	Bosnia and Herzegovina	Any children	Psychosocial support groups in kindergartens	NA	1) kindergarten w/o support groups; 2) no kindergarten	PTSS-10	1) & 2) favourable for experimental condition	–	–	–	–	–	–	–
Thabet et al. (2005)	9–15	111	Gaza	Moderate to severe PTSD reactions	Crisis intervention	7	1) psychoeducation; 2) untreated	CPTSD-RI	0.21 [−0.20, 0.62]	0.32 [−0.19, 0.84]	–	CDI	−0.16 [−0.57, 0.25]	−0.24 [−0.75, 0.27]	–	–
**Uncontrolled pre-post studies**																
Gupta & Zimmer (2008)	8–17	315	Sierra Leone	Any children	Rapid-Ed	9.6	none	IES	–	–	–	–	–	–	–	–
Möhlen et al. (2005)	10–16	13	Kosovo-Albania	Any children	Multimodal Programme	33.6–57.6	none	HTQ	–	–	–	DISYPS-KJ	–	–	–	anxiety: DISYPS-KJ
Onyut et al. (2005)	13–17	6	Somalia	Screened as having PTSD	KIDNET	4.8–14.4	none	CIDI	–	–	–	CIDI	–	–	–	–
Ooi (2012) (pilot)	13–16	4	Africa and Middle East	Mild to severe PTSD symptoms	TRT	9.6	none	–	–	–	–	–	–	–	– –	distress: SDQ (parent) –
Oras et al. (2004)	8–16	13	Mixed	Outpatients	EMDR + psychodynamic therapy	5–25 session (length of session differed for each child)	none	PTSS-C	–	–	–	5 items in the PTSS	–	–		
O’Shea et al. (2000)	ca. 7–11	14	Mixed	Severe psychological problems	Eclectic	5.5	none	–	–	–	–	–	–	–	–	distress: SDQ (teacher)
Pfeiffer and Goldbeck (2017)	14–18	29	Mixed	Mild to severe PTSD symptoms	CBT	12	none	CATS	–	–	–	–	–	–	–	–

NA = not assessed/not available. SMD = standardized mean difference. $ confidence interval estimated from (Bolton et al., 2007). # Dose = total intervention time converted to number of 50 min sessions.

TGI = Traumatic Grief Inventory. SDQ = Strengths and Difficulties Questionnaire. DISYPS-KJ = Diagnostic System for Psychological Disorders. RCMAS = Revised Children’s Manifest Anxiety Scale. YSF = Youth Self Report. SCARED-5 = Screen for Anxiety Related Emotional Disorders. DSRS = Depression Self-Rating Scale. CIDI = Composite International Diagnostic Interview. PTSS = Posttraumatic Stress Symptom Scale. APAI = Acholi Psychosocial Assessment Instrument. CDI = Children’s Depression Inventory. MINI = Mini-International Neuropsychiatric Interview. CRIES-13 = Children’s Revised Impact of Event Scale. IES = Impact of Events Scale. CPSS = Child PTSD Symptom Scale. PTSS-C = Posttraumatic Stress Symptom Scale for Children. HTQ = Harvard Trauma Questionnaire. CPTSD-RI = Child Post Traumatic Stress Reaction Index. CAPS-CA = Clinician Administered PTSD Scale for Children and Adolescents.

TF-CBT = trauma-focused CBT. KIDNET = Narrative Exposure Therapy. CBT = Cognitive Behavioural Therapy. TRT = Teaching Recovery Techniques. CCPT = Child Centred Play Therapy. TST = Trauma Systems Therapy. EMDR = Eye Movement Desensitization and Reprocessing. IPT = Interpersonal Therapy. CP = Creative Play. Rapid Ed = Rapid Educational Response. Med-Relax = meditation/relaxation. WL = waiting list. med = medical care.

Therapeutic effects for traumatic grief, anxiety, and general distress are not presented in Table 1 and thus are presented here. The study of Kalantari and colleagues was the only study on traumatic grief symptoms (Kalantari et al., 2012). The authors used writing for recovery as treatment condition and reported a medium effect compared to untreated controls (SMD = 0.67, 95% CI [0.15, 1.19]). The study of Fazel et al. (2009) resulted in negative treatment effects on general distress using a multilevel counselling approach compared to an untreated ethnic minority group (SMD = −0.67, 95% CI [−1.09, −0.25]). Any other effects on general distress were small and not significant, with the largest effect being reported by Catani et al., 2009, who compared narrative exposure therapy to a meditation-relaxation control group (SMD = 0.34, 95% CI [−0.37, 1.06]). Treatment effects on anxiety symptoms covered the range from SMD = −0.20, 95% CI [−0.56, 0.15] (Lange-Nielsen et al., 2012) to SMD = 0.29, 95% CI [−0.26, 0.84] (Ehntholt et al., 2005), all controls were untreated, not one effect was significant. Ispanovic and colleagues reported only qualitative positive effects of the participation in psychosocial youth club activities on symptoms of anxiety (Išpanović-Radojković et al., 2002).

Altogether, 35 between-group effect sizes were calculated. Six were significant, four of them were positive and two negative. All significant effect sizes were achieved in group treatment settings. Due to the great variety of treatments and small numbers of studies in every group of control conditions, we did not further integrate the effect sizes.

#### 3.2.4. Pre-post effect sizes in all active treatment groups

To detect any sign of an effective treatment, pre-post effect sizes were calculated for any experimental and control condition with an active treatment condition, irrespective of whether the treatment was psychotherapeutic or unspecific (see Table 2). Fourteen of 20 pre-post effect sizes for PTSD were significant, ranging from small (SMC = 0.29, Lange-Nielsen et al., 2012) to large (SMC = 1.94, Onyut et al., 2005). Eight of the significant effects were achieved using CBT techniques, two in a meditation-relaxation condition (Catani et al., 2009; Schauer, 2008), one with a general education programme (Gupta & Zimmer, 2008), one with EMDR combined with psychodynamic therapy (Oras et al., 2004), one with a writing intervention (Lange-Nielsen et al., 2012), and a multilevel treatment particularly oriented to the needs of young refugees (Ellis et al., 2013). Seven of 19 calculated effect sizes for depression were significant, six of them showed positive effects between medium (SMC = 0.31, Tol et al., 2012, in the group of girls) and large (SMC = 1.98, Betancourt et al., 2012a, in the group of girls treated with IPT), with the clinically relevant results being achieved in three CBT conditions, two conditions that used IPT (Betancourt et al., 2012a), one using EMDR (Oras et al., 2004), and one using creative play (Betancourt et al., 2012a). Writing for recovery had negative effects on depression symptoms (SMC = −1.12, Lange-Nielsen et al., 2012), but beneficial effects on traumatic grief symptoms (SMC = 0.96, 95% CI [0.51, 1.41]) (Kalantari et al., 2012) (not reported in Table 2). Thirteen within-group comparisons on general distress were calculated, with eight significant effect sizes, ranging from medium (SMC = 0.40, Dybdahl, 2001) to large (SMC = 1.11, Catani et al., 2009). Five conditions with significant effect sizes used CBT, one a combination psychosocial support and medical care of the mother (Dybdahl, 2001), one meditation-relaxation (Catani et al., 2009), and one was eclectic (O’Shea et al., 2000). Regarding effects on anxiety, two of five effect sizes were significant and medium to large, both in the study of Tol and colleagues (SMC = 0.79 in boys and SMC = 1.06 in girls, Tol et al., 2012, with CBT).

**Table 2.** Pre-post effect sizes (standardized mean changes) by outcome.

			PTSD	Depression	Anxiety	General distress
Study	Treatment (TG)/(CG)	n	SMC [95% CI]	SMC [95% CI]	SMC [95% CI]	SMC [95% CI]
Betancourt et al. (2012a)	IPT, boys (TG)	21	–	1.06 [0.52, 1.60]	–	–
Betancourt et al. (2012a)	IPT, girls (TG)	41	–	1.98 [1.753, 2.40]	–	–
Betancourt et al. (2012a)	CP, boys (TG)	22	–	0.97 [0.45, 1.48]	–	–
Betancourt et al. (2012a)	CP, girls (TG)	31	–	0.11 [−0.26, 0.48]	–	–
Catani et al. (2009)	KIDNET (TG)	16	1.63 [0.96, 2.31]	–	–	1.11 [0.48, 1.73]
Catani et al. (2009)	Med-Relax (CG)	15	1.53 [0.84, 2.22]	–	–	1.08 [0.44, 1.72]
Dybdahl (2001)	Psychosocial support + med (TG)	35^&^	–	0.08 [−0.26, 0.43]	–	0.40 [0.03, 0.77]
Ehntholt et al. (2005)	TRT (TG)	15	0.68 [0.08, 1.27]	0.13 [−0.40, 0.66]	0.30 [−0.25, 0.86]	0.44 [−0.25, 1.14]
Ellis et al. (2013)	TST (TG)	23	0.57$ [0.10, 1.04]	0.20 [−0.24, 0.63]	–	–
Fazel et al. (2009)	Multilevel (TG)	47	–	–	–	0.24 [−0.06, 0.55]
Gupta & Zimmer (2008)	Rapid-Ed (TG)	282	1.12 [0.97, 1.27]	–	–	–
Išpanović-Radojković et al. (2002)	Youth Club (TG)	28	nr	nr	nr	–
Kalantari et al. (2012)	WfR (TG)	29	–	–	–	–
Lange-Nielsen et al. (2012)	WfR (TG)	66	0.29 [0.02, 0.55]	−1.12 [−1.28, −0.96]	−0.08 [−0.56, 0.15]	–
Möhlen et al. (2005)	Multimodal (TG)	10	0.61 [−0.11, 1.33]	0.45 [−0.25, 1.15]	0.36 [−1.42, 2.15]	–
Onyut et al. (2005)	KIDNET (TG)	6^£^	1.94§ [0.76, 3.12]	1.49 [−1.49, 4.47]	–	–
Ooi (2012)	TRT pilot (TG)	4	–	–	–	0.42 [−0.69, 1.53]
Ooi et al. (2016)	TRT (TG)	45	0.67 [0.32, 1.01]	0.43 [0.10, 0.75]	–	parents: 0.41 [0.08, 0.73], teachers: −0.08 [−0.37, 0.21]
Ooi et al. (2016)	TRT, waitlist^#^ (CG)	37	0.17 [−0.17, 0.51]	0.16 [−0.18, 0.50]	–	parents: 0.32 [−0.04, 0.67], teachers: 0.53 [0.16, 0.90]
Oras et al. (2004)	EMDR+PD (TG)	13	1.79 [1.03, 2.56]	1.50 [0.77, 2.24]	–	–
O’Shea et al. (2000)	Eclectic (TG)	7	–	–	–	0.99 [0.06, 1.93]
Pfeiffer and Goldbeck (2017)	CBT (TG)	29	0.85 [0.41, 1.30]	–	–	–
Schauer (2008)	KIDNET (TG)	25	1.55 [1.02, 2.08]	0.13 [−0.28, 0.54]	–	–
Schauer (2008)	Med-Relax (CG)	22	1.67 [1.09, 2.25]	0.10 [−0.33, 0.53]	–	–
Schottelkorb et al. (2012)	CCPT (TG)	14	0.27 [−0.30, 0.83]	–	–	–
Schottelkorb et al. (2012)	TF-CBT (CG)	12	0.21 [−0.39, 0.81]	–	–	–
Šehović (2002)	CBT (TG)	nr	nr	–	–	–
Šestan (2002)	Psychosocial support (TG)	32	nr	–	–	–
Thabet et al. (2005)	CI (TG)	47	0.10 [−0.20, 0.40]	−0.19 [−0.46, 0.09]	–	–
Thabet et al. (2005)	TE (CG)	22	0.10 [−0.34, 0.53]	−0.03 [−0.45, 0.39]	–	–
Tol et al. (2012)	CBT, boys (TG)	122	0.61¶ [0.40, 0.82]	0.36¶ [0.16, 0.56]	0.79¶ [0.58, 1.01]	0.60 [0.39, 0.80]
Tol et al. (2012)	CBT, girls (TG)	76	0.54¶ [0.28, 0.80]	0.31¶ [0.06, 0.56]	1.06¶ [0.78, 1.35]	0.60 [0.34, 0.86]

In the case of non-assessment cells are left empty. nr = not reported.

$ post-assessment three months after the end of the intervention. § calculated from follow-up data. & group sizes unclear due to not indicated missings. # ES also computed for control group, as it was treated and assessed after the waiting period. ¥ ES are not based on post-assessment, but on group-differences in pre- to post-assessment changes. £ For the review and meta-analysis, *n* = 2 missing data at post-assessment were imputed using the follow-up data. ¶ In the study of Tol, the missing group pre-treatment variance was replaced using the variance for the total sample.

TG = Treatment group. CG = Control group. WfR = Writing for Recovery. PD = Psychodynamic Therapy. TF-CBT = trauma-focused CBT. KIDNET = Narrative Exposure Therapy. CBT = Cognitive Behavioural Therapy. TRT = Teaching Recovery Techniques. CCPT = Child Centred Play Therapy. TST = Trauma Systems Therapy. EMDR = Eye Movement Desensitization and Reprocessing. IPT = Interpersonal Therapy. CP = Creative Play. Rapid-Ed = Rapid Educational Response. Med-Relax = meditation/relaxation. WL = waiting list. med = medical care.

### 3.3. Part 2: meta-analysis

Twenty pre-post effect sizes for PTSD and 19 for depression were the basis for calculation of a combined effect. The combined pre-post effect size for PTSD was SMC = 0.78, 95% CI [0.53, 1.03], and for depression SMC = 0.35, 95% CI [0.04, 0.67]. The overall test suggested considerable heterogeneity among the true effects for both PTSD (I^2^ = 88.6%, Q = 129.0, *df* = 19, *p* < .05) and depression (I^2^ = 93.1%, Q = 349.2, *df* = 18, *p* < .05), revealing that the combined effect cannot be interpreted meaningfully. Residence status (refugee or internally displaced) was not a significant moderator (PTSD: QM = 0.39, *p* = .53; depression: QM = 0.30, *p* = .58). Indication for publication bias was not tested as all known tests perform poorly in the presence of substantial heterogeneity (Peters et al., 2010).

**Figure 2.**
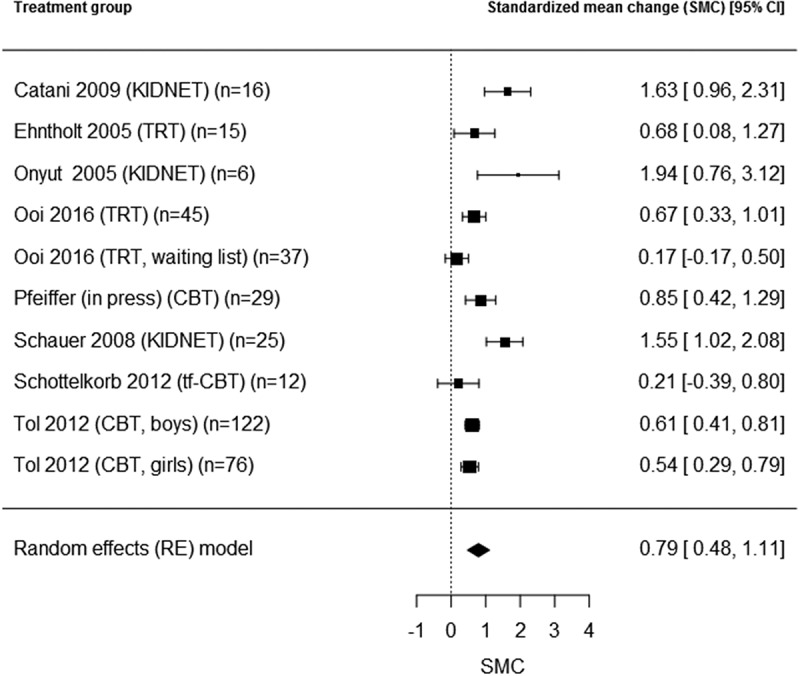
PTSD pre-post effects (SMC) in groups with cognitive behavioural treatment.

**Figure 3.**
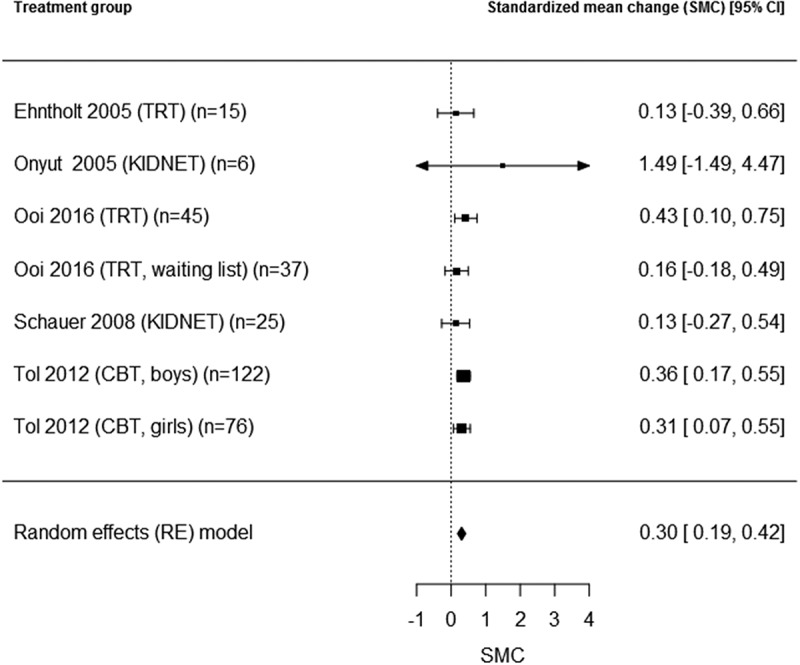
Depression pre-post effects (SMC) in groups with cognitive behavioural treatment.

We repeated the meta-analysis for the subgroup of CBT-based interventions, as we expected this group to be more homogeneous. PTSD outcomes were reported for 10 active CBT treatment groups (Catani et al., 2009; Ehntholt et al., 2005; Onyut et al., 2005; Ooi et al., 2016; both treatment and later treated waitlist group; Pfeiffer & Goldbeck, 2017; Schauer, 2008; Schottelkorb et al., 2012; Tol et al., 2012; separately for boys/girls), and depression outcomes for seven active CBT treatment groups (Ehntholt et al., 2005; Onyut et al., 2005; Ooi et al., 2016; treatment and later treated waitlist group; Schauer, 2008; Tol et al., 2012; separately for boys/girls). The combined pre-post effect sizes were SMC = 0.79, 95% CI [0.47, 1.11] for PTSD (total *N* = 394), and SMC = 0.30, 95% CI [0.18, 0.43] for depression (total *N* = 337) (Figures 2 and 3, respectively). The overall test for heterogeneity was significant for PTSD (I^2^ = 83.5, Q = 35.8, *df* = 9, *p* < .05), but not for depression (I^2^ = 0.0, Q = 3.3, *df* = 6, *p* = .77), indicating a meaningful combined effect size for depression. Residence status (refugee or internally displaced) was not a significant moderator (PTSD: QM = 1.3, *df* = 1, *p* = .25; depression: QM = 0.09, *df* = 1, *p* = .77). A regression test for funnel plot asymmetry on the homogeneous effect size for depression did not indicate any publication bias (*p* = .32).

## 4. Discussion

In our review on treatments for displaced minors who were traumatized by war, we considered data from published and unpublished studies and did not exclude non-randomized trials. Our goal was to include any possibly effective interventions at this early stage of treatment research. In spite of our very broad search criteria, we were able to find only 23 studies. The results demonstrate that treatment studies in displaced minors constitute a relatively young field of research. All identified studies were published in the last 15 years, with a substantial number of pilot studies and studies of poor methodological quality. Very diverse treatments were applied, most of them not evidence-based, and a substantial number of them did not yield significant effects. Before discussing this further we should consider the limitations of this paper.

### 4.1. Limitations

First, some of the original studies had methodological flaws which affected the reported statistics in diverse directions. Only a minority of studies used randomized controlled designs. Some authors reported serious problems in motivating the parents to participate, or changes in the residence status that prevented the children from attending all therapy sessions. Almost all studies relied on completer analyses, with sometimes high attrition rates and no reported reasons for drop-out. These shortcomings, together with others like no blinding of raters, might have led to overestimation or underestimation of the true effect sizes.

Second, there are limitations on the level of the narrative review. Between-group effect sizes were not comparable, as even randomized controlled trials control for diverse variables which have a substantial impact on the treatment effect (e.g. gender).

Third, there are limitations on the level of the meta-analysis of the pre-post effect sizes. We included uncontrolled studies in order to detect any possibly effective treatment, and the computed SMCs certainly include time effects. So, the one non-heterogeneous overall effect for the treatment of depressive symptoms with CBT is probably overestimating the true effect.

### 4.2. Treatment effects

Most between-group effect sizes in war-affected displaced minors were not significant, some were negative, although several trials applied evidence-based psychotherapies. Concerning PTSD symptoms, only two studies using CBT detected a clinically relevant difference between the treatment group and untreated controls. The analysis of pre-post effects confirmed the positive effects for CBT, but there were some other treatments with positive pre-post effects that might be promising candidates for future investigation: EMDR, meditation-relaxation, an educational programme in schools as developed by the UNESCO, a stepped systemic treatment designed to the needs of young refugee youth, and writing for recovery. The positive CBT and EMDR effects are in line with the recent literature on youth from the general population (Gillies, Taylor, Gray, O’Brien, & D’Abrew, 2013; Gutermann et al., 2016). The mean pre-post effect size in our study cannot be meaningfully interpreted due to the high heterogeneity, which has also been reported in a number of other meta-analyses (e.g. Morina et al., 2017). In our view this shows how understudied this field of research is, as there are too few studies with obviously too different interventions available. Effect sizes in our sample were somewhat lower than effect sizes from the general population (Gillies et al., 2013: SMD = 1.34 compared to any control condition; Gutermann et al., 2016: SMC = 0.89 and SMD = 0.89 compared to untreated controls; Morina et al., 2016: SMD = 0.83 compared to waitlist controls), although they probably overestimated true treatment effects. They were also lower than those reported in children affected by war (Morina et al., 2017: SMC = 1.15). Part of the difference might be accounted by the small overlap between Morina et al. and our study. First, Morina and colleagues only included RCTs and the overlap with the eight RCTs in our study involves only three publications (Catani et al., 2009; Schauer, 2008; Tol et al., 2012). This is explained by differing inclusion criteria: Morina et al. included child soldiers, restricted their analysis on those who live in low- and middle-income countries, and did not include those who had to flee to a high-income country. However, some part of the difference in effect sizes might be no artefact: displaced children in need of psychosocial treatment might benefit substantially less from available treatments due to a number of factors, like loss of social support, and their specific living conditions might account in some part for the lower effects in our study. As we did not find any indication that residence status (refugees or internally displaced) had some impact on treatment benefit, this could hold true for both groups.

Our results for depression as outcome reveal a similar lack of evidence. Compared to untreated controls, only IPT was proven to be beneficiary out of all investigated treatments. Significant pre-post effect sizes were achieved using established interventions like CBT, IPT, and EMDR. This, again, is in line with the meta-analysis of Gillies et al. (2013), who showed that exposure-based interventions in children and adolescents from the general population had larger effects on depression symptoms than other psychological approaches. CBT treatments resulted in an overall small to medium pre-post effect size for depressive symptoms (SMC = 0.30) in this group, which is lower than psychotherapy effects in general population minors with mixed traumata (Gillies et al., 2013: SMD = 0.80 compared to any control condition; Gutermann et al., 2016: SMC = 0.62; Morina et al., 2016: SMD = 0.30 compared to waitlist controls).

It is worth noting that in our review several significant effects resulted from treatments that were provided by trained lay persons (Betancourt et al., 2012a; Catani et al., 2009; Dybdahl, 2001; Gupta & Zimmer, 2008; Pfeiffer & Goldbeck, 2017; Schauer, 2008; Tol et al., 2012), demonstrating that substantial symptom reduction can be achieved by this means. On the other hand, some treatments delivered by clinicians were ineffective (Schottelkorb et al., 2012; Thabet et al., 2005) or even harmful (Lange-Nielsen et al., 2012). Some treatments were effectively administered in group settings (Betancourt et al., 2012a; Pfeiffer & Goldbeck, 2017) or at school (Gupta & Zimmer, 2008; Ooi et al., 2016; Schauer, 2008; Tol et al., 2012). Other reviews (Newman et al., 2014; Rolfsnes & Idsoe, 2011) have reported moderate (Rolfsnes & Idsoe, 2011) to large (Newman et al., 2014) effect sizes for trauma-related psychotherapies in the group/school setting, even if they were provided by trained lay persons like teachers or social workers. A substantial number of affected school-aged refugee children could be reached this way, if effective interventions were available. The potential of dissemination of evidence-based treatments by training lay practitioners should be further investigated.

To summarize, we saw that therapeutic effects for war-affected displaced minors stay behind the expected range, which is especially discouraging given the fact that they often decrease on the long run, even one month post-treatment (Gillies et al., 2016, 2013; Schauer, 2008). Additionally, an overall zero effect indicates that, while some subjects might benefit from a treatment, it probably has negative effects on others; a fact that has been discussed in the past (Ertl & Neuner, 2014; Tol et al., 2014). We think that displaced children constitute a particularly vulnerable group with specific challenges for therapists. Perhaps available interventions need to be adapted to the specific needs of this population and the specific context factors in this group, as even CBT-based interventions showed only moderate effect sizes. The population is certainly understudied, which is deplorable in light of their number. Hence, more large quality studies are urgently needed for concrete treatment recommendations.
